# The relationship between weight gain during chemotherapy and outcomes in patients with advanced non‐small cell lung cancer

**DOI:** 10.1002/jcsm.13426

**Published:** 2024-03-11

**Authors:** Eric J. Roeland, Florian J. Fintelmann, Fiona Hilton, Ruoyong Yang, Ed Whalen, Lisa Tarasenko, Roberto A. Calle, Philip D. Bonomi

**Affiliations:** ^1^ Knight Cancer Institute Oregon Health and Science University Portland OR USA; ^2^ Department of Radiology Massachusetts General Hospital Boston MA USA; ^3^ Pfizer Inc. Groton CT USA; ^4^ Pfizer Inc. New York NY USA; ^5^ Pfizer Worldwide Research and Development Cambridge MA USA; ^6^ Rush University Medical Center Chicago IL USA

**Keywords:** cachexia, non‐small cell lung cancer, survival, weight, weight gain

## Abstract

**Background:**

This post hoc, pooled analysis examined the relationship between different weight gain categories and overall survival (OS) in patients with non‐small cell lung cancer (NSCLC) receiving first‐line platinum‐based chemotherapy.

**Methods:**

Data were pooled from the control arms of three phase III clinical studies (NCT00596830, NCT00254891, and NCT00254904), and the maximum weight gain in the first 3 months from treatment initiation was categorised as >0%, >2.5%, and >5.0%. Cox proportional hazard modelling of OS was used to estimate hazard ratios (HRs) for each category, including baseline covariates, time to weight gain, and time to confirmed objective response (RECIST Version 1.0).

**Results:**

Of 1030 patients with advanced NSCLC (IIIB 11.5% and IV 88.5%), 453 (44.0%), 252 (24.5%), and 120 (11.7%) experienced weight gain from baseline of >0%, >2.5%, and >5.0%, respectively. The median time to weight gain was 23 (>0%), 43 (>2.5%), and 45 (>5.0%) days. After adjusting for a time‐dependent confirmed objective response, the risk of death was reduced for patients with any weight gain (>0% vs. ≤0% [HR 0.71; 95% confidence interval—CI 0.61, 0.82], >2.5% vs. ≤2.5% [HR 0.76; 95% CI 0.64, 0.91] and >5.0% vs. ≤5.0% [HR 0.77; 95% CI 0.60, 0.99]). The median OS was 13.5 versus 8.6 months (weight gain >0% vs. ≤0%), 14.4 versus 9.4 months (weight gain >2.5% vs. ≤2.5%), and 13.4 versus 10.2 months (weight gain >5.0% vs. ≤5.0%).

**Conclusions:**

Weight gain during treatment was associated with a reduced risk of death, independent of tumour response. The survival benefit was comparable for weight gain >0%, >2.5%, and >5.0%, suggesting that any weight gain may be an early predictor of survival with implications for the design of interventional cancer cachexia studies.

## Introduction

Cancer cachexia is a multifactorial syndrome frequently characterised by anorexia, unintentional weight loss (including skeletal muscle loss), fatigue, functional impairment, poor quality of life, and worse survival.^1^, ^2^, ^3^ The pathogenesis of cancer cachexia is under investigation and involves abnormal metabolism and weight loss that cannot be fully reversed with nutritional supplementation.^2^, ^4^ Cachexia is frequently observed across various cancers,^5^ and its management is challenging and, ideally, should include a multimodal, multidisciplinary team‐based approach.^3^, ^6^, ^7^ Despite multiple clinical trials evaluating novel pharmacological interventions to treat cancer cachexia,^8^ evidence‐based guidelines do not strongly endorse any specific drug.^3^, ^6^ Moreover, regulatory agencies across the United States and Europe have not approved any drug to treat cancer cachexia.

Defined weight loss over time is a common inclusion criterion and endpoint amongst cancer cachexia studies. In fact, data demonstrate that ~20% of patients with non‐small cell lung cancer (NSCLC) experience 5–10% weight loss, and 15% experience >10% weight loss over the preceding 6 months.^9^ In advanced NSCLC, the increasing severity of cachexia is associated with fewer completed chemotherapy cycles^10^ and reduced survival.^11^, ^12^ Given the incidence of weight loss in patients with advanced NSCLC, this cancer histology has been the focus of most cancer cachexia randomised controlled trials. For example, the largest modern interventional cancer cachexia study in over 900 patients required that patients with NSCLC should have ≥5% weight loss over the previous 6 months (or body mass index [BMI] <20 kg/m^2^) to participate.^13^

Conversely, weight stabilisation or weight gain during cancer treatment is associated with increased survival.^14^, ^15^, ^16^, ^17^ For example, a retrospective analysis found that patients with advanced, non‐squamous NSCLC and weight gain >5.0% during treatment were associated with improved overall survival (OS) and progression‐free survival (PFS). Interestingly, the association was also apparent for any degree of weight gain (i.e., >0%).^18^ Therefore, weight gain may serve as an early indicator of the clinical benefit of cancer therapy; yet, the degree of weight gain over time remains understudied, and investigators require early cut‐off values that indicate a clinically meaningful value of weight gain. Most interventional NSCLC clinical trials assess treatment response based on a change in longitudinal tumour measurements (i.e., the National Cancer Institute's Response Evaluation Criteria in Solid Tumors [RECIST]^19^), with the first radiological assessment occurring at ~3 months. Therefore, this post hoc analysis aimed to evaluate the frequency of maximum weight gain in patients with NSCLC receiving first‐line platinum‐based chemotherapy at different percentage cut‐off values within 3 months of starting treatment and to evaluate their relationship with OS and PFS. We hypothesised that weight gain during the first four cycles of treatment (~3 months) might have prognostic significance and that an alternative to the 5% cut‐off value may exist.

## Methods

### Patients and study design

Data were pooled from the control arms of three phase III international randomised, open‐label clinical studies (NCT00596830, NCT00254891, and NCT00254904) conducted between November 2005 and March 2011. In all three studies, adult patients (≥18 years old) with advanced NSCLC received first‐line standard‐of‐care (SOC) platinum‐based chemotherapy in the control arms. The studies were conducted in compliance with the ethical principles originating in or derived from the Declaration of Helsinki and in compliance with all International Conference on Harmonisation Good Clinical Practice Guidelines. All participants provided written informed consent.

The key inclusion criteria across all three studies included a diagnosis of advanced NSCLC (stage IIIB [with pleural effusion] or stage IV); no previous systemic treatment for NSCLC with chemotherapy, immunotherapy, biological response modifiers or other investigational drugs (NCT00596830 permitted adjuvant chemotherapy completed ≥1 year before randomisation, and all studies permitted prior surgery or radiation therapy if completed ≥3 weeks before randomisation); and an Eastern Cooperative Oncology Group (ECOG) performance status of 0–1.^20^

Patients were excluded if they had small‐cell or carcinoid lung cancer (and all other active cancer types for NCT00596830), known central nervous system metastasis (all studies) or a pre‐existing autoimmune or antibody‐mediated disease (NCT00254891 and NCT00254904). Patients were also excluded from NCT00596830 if they required chronic corticosteroid use (within 1 week prior to treatment) or had a history of uncontrolled diabetes (HbA1c > 8.0%).

### Treatment

Patients in the control arms of the three studies received standard platinum‐based doublet chemotherapy consisting of paclitaxel and carboplatin (NCT00254891 and NCT00596830) or gemcitabine and cisplatin (NCT00254904), and each was considered SOC at the time. Chemotherapy was administered in 3‐week cycles for a maximum of six cycles.

### Assessments

Baseline and clinical characteristics were collated, including age, sex, BMI, race, smoking status, ECOG performance status, NSCLC type, and disease stage.

#### Weight

During the three studies, investigators recorded weight at baseline and prior to dosing on the first day of each 3‐week treatment cycle (approximately Days 1, 22, 43, 64, 85, and 106 after completing all six cycles). There was an additional post‐treatment weight measurement at 3 weeks after the last treatment cycle.

For each patient, all weight measurements following 90 days of starting treatment were included in the analyses, regardless of planned or unplanned visits. Any measurements recorded after initiating a new cancer‐directed therapy were excluded.

The percentage weight change from baseline was calculated for each post‐baseline time point, and the maximum percentage weight gain over the first 90 days from treatment initiation was determined. Each patient was categorised, based on maximum percentage weight gain in the first 90 days from the start of treatment, using cut‐points of (A) ≤0% versus >0%, (B) ≤2.5% versus >2.5%, and (C) ≤5.0% versus >5.0%. The day was determined on which percentage weight gain exceeded 0%, 2.5%, or 5.0% (by any amount, not necessarily the maximum) within the first 90 days since starting treatment.

#### Confirmed objective response rate

The investigator‐assessed confirmed objective response rate was based on RECIST^19^ Version 1.0. Complete response (CR) was defined as the complete disappearance of all target lesions and non‐target disease with no new lesions. Partial response (PR) was defined as a ≥30% decrease from the baseline of the sum of the longest diameters of all measurable target lesions. Confirmed responses were those that persisted ≥4 weeks after the initial objective response documentation. Each patient was categorised as a responder (CR or PR) or non‐responder (stable disease or progression).

#### Survival

OS was defined as the time from treatment initiation to death due to any cause, censored on the date of the last contact for patients who were alive. PFS was the time from the treatment's start to the date of the first documented disease progression or death due to any cause, whichever occurred first, censored for each study.

### Statistical analyses

These post hoc analyses utilised pooled data from the control arms of three phase III international, randomised, open‐label clinical studies (NCT00596830, NCT00254891, and NCT00254904). Patients eligible for analyses included those who received at least one dose of SOC treatment and had a pre‐treatment weight measurement, at least one post‐treatment weight measurement and available efficacy data (survival and/or objective response).

For all multivariate analyses, modelling was replicated using two sets of covariates. Two models were used because the relationship is unknown. Model I included age (<65 and ≥65 years), sex (male and female), baseline BMI (<20 and ≥20 kg/m^2^), race (Asian and non‐Asian), smoking (never and ever), baseline ECOG performance status (0 and 1), adenocarcinoma (yes and no) and disease stage (IIIB and IV), all as categorical variables. Model II included the covariates of age, baseline BMI, and baseline weight as continuous variables and all others (sex, race, smoking, baseline ECOG performance status, adenocarcinoma, and disease stage) as categorical variables. The two models treat continuous variables differently, with the advantage of one model over the other depending on the relationship (linear vs. non‐linear) between the continuous variables and log hazard ratio (HR).

Multivariate logistic regression was used to fit baseline covariates to the response variable of weight gain, with stepwise regression to select the covariates associated with the response variable of weight gain. The only variables considered were the a priori selected candidate variables outlined in the preceding paragraph. For continuous variables, a generalised additive model was fitted to explore the linearity of any relationship with weight gain.

Time‐to‐event endpoints of OS and PFS were summarised using the Kaplan–Meier method. The median time to event with 95% confidence intervals (CIs) was estimated using the Brookmeyer and Crowley method.^21^ A stepwise Cox proportional hazard model was applied to select covariates associated with the response variable, with estimates of HRs and 95% CIs for each covariate. Additional analyses were conducted using Cox proportional hazard models that included (1) weight gain as a time‐dependent variable and (2) weight gain and objective response as time‐dependent variables. Any associations between OS or PFS and weight gain were adjusted for baseline and clinical characteristic covariates.

Separate interaction analyses were performed to determine if either age or tumour response (CR and PR vs. stable disease and progression) had an impact on survival independent of weight gain. A proportional hazard model with a time‐dependent variable was forced to include the covariate of interest (tumour response or age) in Models I and II. Stepwise regression was used to select other significant baseline covariates.

Receiver operating characteristic (ROC) curve analyses, conducted also under the proportional hazard model, were performed to identify the potentially best predictive weight gain cut‐point for survival at 6, 12, and 24 months.

All analyses were conducted using SAS Version 9.4 (SAS Institute Inc., Cary, NC). We report HRs and 95% CIs rather than *P* values, as the former are more appropriate for post hoc analyses. A 95% CI that does not include 1 suggests an association between the variables of interest.

## Results

### Baseline characteristics

**Figure 1:**
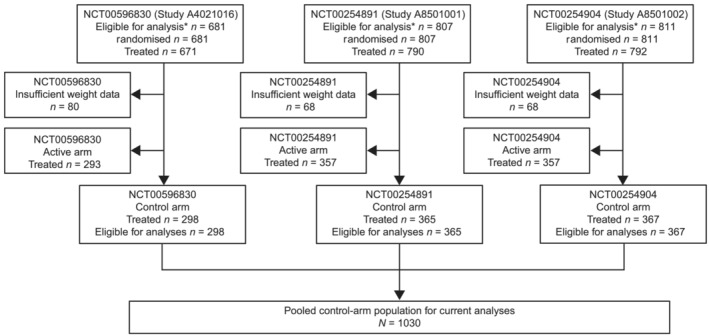
Patient disposition for current analysis. *Eligibility for analysis following informed consent review.

Across the three studies, a total of 1030 patients were eligible for the current analyses (*Figure* *1*). Most had stage IV NSCLC (88.5%) and were predominantly male (70.5%) with a mean age of 60.9 years and a mean BMI of 24.6 kg/m^2^ (*Table* *1*). Baseline characteristics were similar across the three studies, except for NCT00596830, in which no patients had adenocarcinoma at baseline (vs. ~50% of patients in NCT00254891 and NCT00254904), consistent with exclusion criteria.

**Table 1:** Baseline and clinical characteristics

Characteristic	NCT00596830 (*N* = 298)	NCT00254891 (*N* = 365)	NCT00254904 (*N* = 367)	Pooled patient group (*N* = 1030)
Age (years)				
Mean (SD)	61.3 (9.0)	61.5 (9.6)	59.9 (9.6)	60.9 (9.4)
Median (range)	62 (36–83)	62 (34–87)	60 (34–85)	62 (34–87)
Age, *n* (%)				
<65 years	185 (62.1)	218 (59.7)	239 (65.1)	642 (62.3)
≥65 years	113 (37.9)	147 (40.3)	128 (34.9)	388 (37.7)
Race, *n* (%)^a^				
Asian	53 (17.8)	44 (12.1)	75 (20.4)	172 (16.7)
Non‐Asian	245 (82.2)	320 (87.9)	292 (79.6)	857 (83.3)
Sex, *n* (%)				
Male	228 (76.5)	240 (65.8)	258 (70.3)	726 (70.5)
Baseline BMI (kg/m^2^)				
Mean (SD)	24.3 (4.5)	24.9 (4.7)	24.4 (3.9)	24.6 (4.4)
Median (range)	24.1 (14.7–40.9)	24.3 (14.8–42.4)	24.2 (12.4–36.4)	24.2 (12.4–42.4)
Baseline BMI, *n* (%)^b^				
<20 kg/m^2^	54 (18.1)	48 (13.3)	49 (13.4)	151 (14.7)
≥20 kg/m^2^	244 (81.9)	314 (86.7)	317 (86.6)	875 (85.3)
Adenocarcinoma, *n* (%)^c^				
Yes	0 (0.0)	207 (56.7)	173 (47.1)	380 (36.9)
No	298 (100.0)	158 (43.3)	194 (52.9)	650 (63.1)
Baseline ECOG PS, *n* (%)^d^				
0	107 (36.8)	133 (36.7)	118 (32.2)	358 (35.1)
1	184 (63.2)	229 (63.3)	248 (67.8)	661 (64.9)
Baseline cancer stage, *n* (%)^e^				
IIIB	33 (11.1)	47 (12.9)	38 (10.4)	118 (11.5)
IV	265 (88.9)	318 (87.1)	329 (89.6)	912 (88.5)
Smoking status, *n* (%)^f^				
Never	31 (10.4)	52 (14.2)	58 (15.8)	141 (13.7)
Ever	267 (89.6)	313 (85.8)	309 (84.2)	889 (86.3)

Abbreviations: BMI, body mass index; ECOG PS, Eastern Cooperative Oncology Group performance score.

^a^ One patient with missing data (pooled sample size = 1029). Asian versus non‐Asian categorisation was selected as the levels of the covariate due to general differences in body weight and the impact this might have on potential weight gain in these studies.

^b^ Four patients with missing data (pooled sample size = 1026).

^c^ Based on primary tumour histology diagnosis; the diagnosis is primarily adenocarcinoma, squamous cell carcinoma, large cell carcinoma, or other. One study excluded patients with adenocarcinoma.

^d^ Eleven patients with missing data (pooled sample size = 1019). ECOG is a 6‐point scale describing a patient's level of independent functioning, with 0 denoting *completely independent/fully active* and 4 denoting *completely disabled* (5 denotes *death*).

^e^ Stage IIIB with pleural effusion (locally advanced) or stage IV (metastatic) based on the American Joint Committee on Cancer classification.

^f^ Ever includes ex‐smokers and current smokers.

### Weight gain

In the first 90 days from treatment initiation, 453 (44.0%), 252 (24.5%), and 120 (11.7%) patients experienced weight gain from baseline of >0%, >2.5%, and >5.0%, respectively (*Table* *2*). The median time to >0%, >2.5%, and >5.0% weight gain was 23, 43, and 45 days, respectively (*Table* *2*).

**Table 2:** Body weight changes in the first 90 days since the start of treatment

Characteristic	Pooled patient group (*N* = 1030)
>0% weight gain, *n* (%)	
Maximum weight gain ≤0%	577 (56.0)
Maximum weight gain >0%	453 (44.0)
Time to first weight gain >0% (days), median (Q1, Q3)	23.0 (22.0, 43.0)
>2.5% weight gain, *n* (%)	
Maximum weight gain ≤2.5%	778 (75.5)
Maximum weight gain >2.5%	252 (24.5)
Time to first weight gain >2.5% (days), median (Q1, Q3)	43.0 (22.5, 63.0)
>5.0% weight gain, *n* (%)	
Maximum weight gain ≤5.0%	910 (88.4)
Maximum weight gain >5.0%	120 (11.7)
Time to first weight gain >5.0% (days), median (Q1, Q3)	45.0 (40.5, 64.0)

Abbreviation: (Q1, Q3), inter‐quartile range.

Across all three categories of weight gain, younger patients (<65 years old) were more likely to gain weight compared with older patients (≥65 years old) (*Table* *S1* [Model I]); however, the relationship was not always linear (*Table* *S1* [Model II]). Other covariates were associated with weight gain but with less consistency across weight gain categories (*Table* *S1*).

### Overall survival

OS was similar across the three studies (data not shown), with median survival ranging from 10.5 to 11.1 months. Generally, a reduced risk of death was associated with never (compared with ever) smoking, a better performance status (ECOG 0 vs. 1), and less advanced disease (stage IIIB vs. IV), whereas an increased risk of death was associated with male sex and a lower BMI (*Table* *S2* [Models I and II]).

#### Overall survival by weight gain

**Figure 2:**
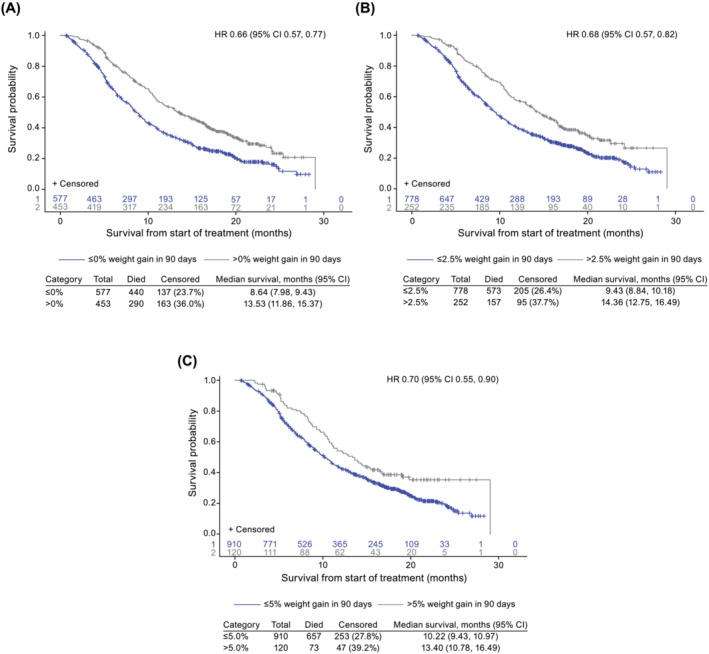
Kaplan–Meier curves of overall survival by weight gain category (A) ≤0% versus >0%, (B) ≤2.5% versus >2.5%, and (C) ≤5.0% versus >5.0%. CI, confidence interval; HR, hazard ratio.

OS for each of the three weight gain categories is shown in *Figure* *2* with analyses in *Table* *S3* (Models I and II). After adjusting for baseline and clinical characteristics, the risk of death was smaller for the >0% versus ≤0% weight gain category (HR 0.66; 95% CI 0.57, 0.77) with an observed median OS of 13.5 versus 8.6 months, respectively (*Figure* *2* *A* and *Table* *S3* [Model I]). Similarly, for the >2.5% versus ≤2.5% weight gain category, the HR was 0.68 (95% CI 0.57, 0.82) with an observed median OS of 14.4 versus 9.4 months, respectively (*Figure* *2* *B* and *Table* *S3* [Model I]). For the >5.0% versus ≤5.0% weight gain category, the HR was 0.70 (95% CI 0.55, 0.90) with an observed median OS of 13.4 versus 10.2 months, respectively (*Figure* *2* *C* and *Table* *S3* [Model I]). When using the individual percentage weight gain for each time point in the modelling, weight gain was associated with a reduced risk of death at any time point (*Table* *S3* [Models I and II]).

After adjusting for the time‐dependent variable of weight gain, there remained associations between baseline and clinical covariates and OS that were similar to those reported above (*Table* *S3* [Models I and II]).

#### Overall survival by weight gain and objective response

**Figure 3:**
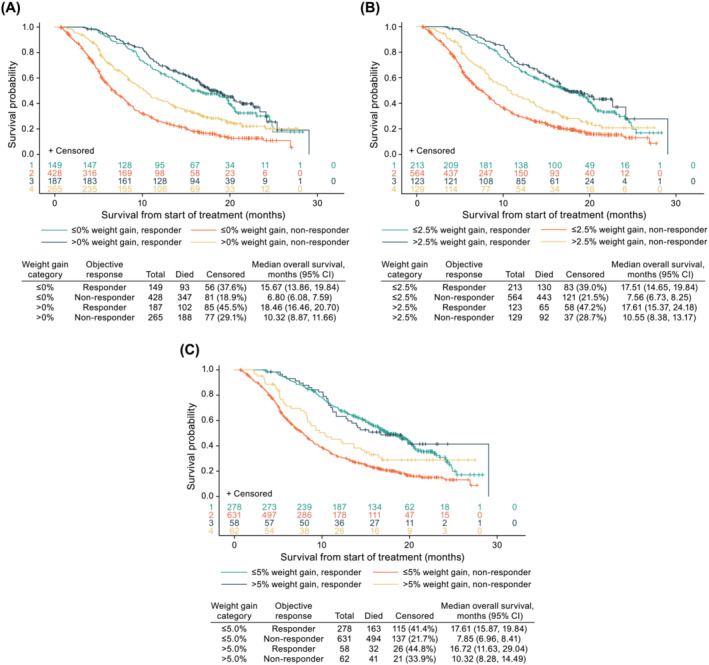
Kaplan–Meier curves of overall survival by weight gain category (A) ≤0% versus >0%, (B) ≤2.5% versus >2.5% and (C) ≤5.0% versus >5.0% and confirmed objective response (complete and partial responses vs. no response). CI, confidence interval.

Weight gain was associated with favourable effects on OS in responders and non‐responders (*Figure* *3* *A*–*C*). The main effects of weight gain and objective response were observed for each weight gain category, with an interaction observed only for the 0% weight gain category (not the 2.5% or 5.0% weight gain categories). The risk of death was smaller for responders in all weight gain categories: >0% (HR 0.45; 95% CI 0.38, 0.54), >2.5% (HR 0.46; 95% CI 0.38, 0.54), and >5.0% (HR 0.45; 95% CI 0.38, 0.53) (*Table* *S4* [Model I]). Additionally, after adjusting for a time‐dependent confirmed objective response, the risk of death was smaller for patients with weight gain >0% versus ≤0% (HR 0.71; 95% CI 0.61, 0.82), >2.5% versus ≤2.5% (HR 0.76; 95% CI 0.64, 0.91) and >5.0% versus ≤5.0% (HR 0.77; 95% CI 0.60, 0.99) (*Table* *S4* [Model I]).

A similar pattern of associations between baseline covariates and OS was observed after adjusting for the time‐dependent variables of weight gain and objective response, with the addition of adenocarcinoma being associated with a reduced risk of death (*Table* *S4* [Models I and II]).

#### Interaction analyses

Analyses were conducted to determine if age or tumour response had an impact on survival independent of weight gain. None of the interactions (weight gain by age or weight gain by objective response) for each weight gain category in Model I or II were statistically significant (*P* > 0.05; data not shown).

#### Receiver operating characteristic curve analyses

The area under the ROC curve conducted at 6, 12, and 24 months was 0.63, 0.61, and 0.60, respectively. At each time point, sensitivity increased while specificity decreased across each of the three weight gain categories. Thus, it was not possible to identify the best predictive weight gain cut‐off value.

### Progression‐free survival

PFS was similar across the three studies, with median PFS ranging from ~4.9 to 5.3 months. A reduced risk of disease progression or death was associated with never smoking, a better performance status, and less advanced disease, whereas Asian patients were at increased risk (*Table* *S5* [Models I and II]).

#### Progression‐free survival by weight gain

**Figure 4:**
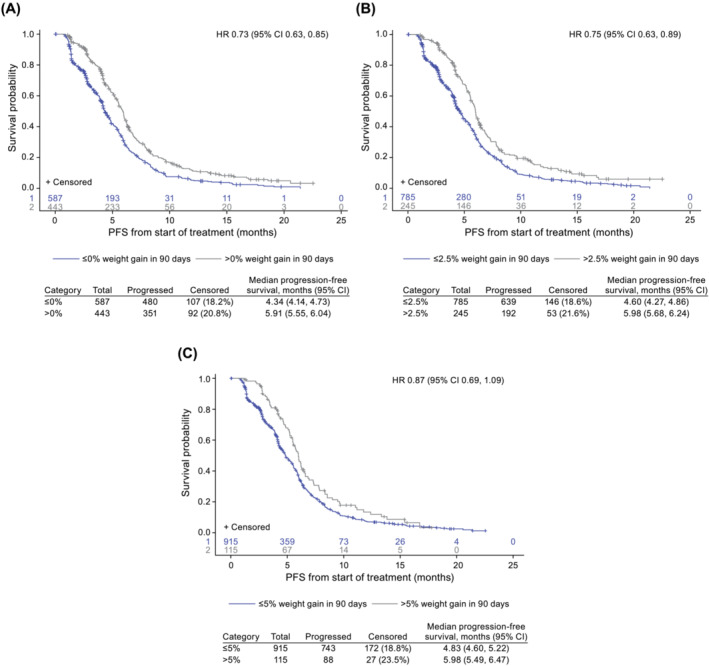
Kaplan–Meier curves of progression‐free survival (PFS) by weight gain category (A) ≤0% versus >0%, (B) ≤2.5% versus >2.5% and (C) ≤5.0% versus >5.0%. CI, confidence interval; HR, hazard ratio.

The risk of disease progression or death was smaller for the >0% versus ≤0% weight gain category (HR 0.73; 95% CI 0.63, 0.85) with an observed median PFS of 5.9 versus 4.3 months, respectively (*Figure* *4* *A* and *Table* *S6* [Model I]) and for the >2.5% versus ≤2.5% weight gain category (HR 0.75; 95% CI 0.63, 0.89) with an observed median PFS of 6.0 versus 4.6 months, respectively (*Figure* *4* *B* and *Table* *S6* [Model I]). The risk of disease progression or death was not observed with weight gain for the >5.0% versus ≤5.0% weight gain category (HR 0.87; 95% CI 0.69, 1.09) with an observed median PFS of 6.0 versus 4.8 months, respectively (*Figure* *4* *C* and *Table* *S6* [Model I]). When the individual percentage weight gain for each time point was used in the modelling, weight gain was also associated with a reduced risk of progression or death at any time (*Table* *S6* [Models I and II]). Associations between baseline covariates and PFS (after adjusting for the time‐dependent variable of weight gain) were similar to those observed with OS (*Table* *S6* [Models I and II]). However, unlike OS, there was an increased risk of disease progression or death for those patients who were younger or Asian, and being male was not associated with greater risk.

#### Progression‐free survival by weight gain and objective response

**Figure 5:**
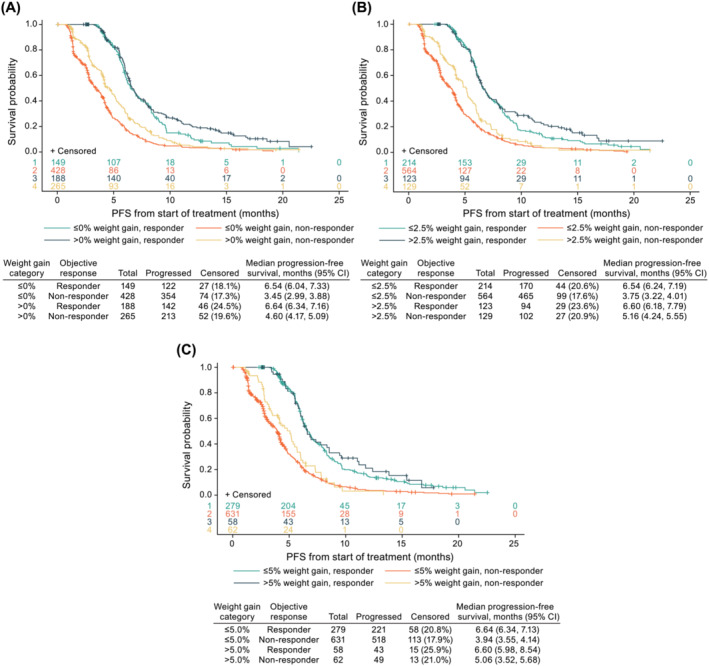
Kaplan–Meier curves of progression‐free survival (PFS) by weight gain category (A) ≤0% versus >0%, (B) ≤2.5% versus >2.5% and (C) ≤5.0% versus >5.0% and confirmed objective response (complete and partial responses vs. no response). CI, confidence interval.

Weight gain was associated with favourable effects on PFS in responders and non‐responders (*Figure* *5* *A*–*C*). A statistical interaction between weight gain and objective response was not observed for any weight gain category. The risk of progression or death was consistently lower for responders in each weight gain category: >0% (HR 0.50; 95% CI 0.42, 0.58), >2.5% (HR 0.50; 95% CI 0.43, 0.58), and >5.0% (HR 0.49; 95% CI 0.42, 0.57) (*Table* *S7* [Model I]). After adjusting for a time‐dependent confirmed objective response, the risk of death was smaller for patients with weight gain >0% versus ≤0% (HR 0.77; 95% CI 0.67, 0.89) and >2.5% versus ≤2.5% (HR 0.85; 95% CI 0.72, 1.00) (*Table* *S7* [Model I]). As with the previous analysis on PFS by weight gain, there were similar associations between baseline covariates and PFS after adjusting for time‐dependent variables of weight gain and objective response (*Table* *S7*).

## Discussion

In this pooled analysis of three multinational clinical trials of patients with advanced NSCLC, weight gain during the first 3 months of starting SOC platinum‐doublet chemotherapy was associated with a reduced risk of disease progression and death, independent of tumour response per RECIST. This 3‐month time frame was clinically meaningful as it aligned with the first radiological assessment. The survival benefit, including OS and PFS, was generally comparable for weight gain >0%, >2.5%, and >5.0%. Collectively, the results of the present study confirm the benefits and prognostic value of weight gain in patients with advanced NSCLC, and we expand upon previous findings^14^, ^15^, ^16^, ^17^, ^18^ by examining three specific weight gain categories and limiting the weight change analysis to the first 3 months of treatment.

Determining a clinically meaningful cut‐off value for percent weight gain has implications for the design of cancer cachexia studies. Although the results of the ROC curve analyses were not sufficiently robust to identify the best predictive value of weight gain, the survival analyses suggest that any weight gain has an impact on survival. Although weight gain may be an ideal cancer cachexia intervention goal, weight stability (or weight loss mitigation) in patients with NSCLC receiving chemotherapy in a hypercatabolic metabolic state is meaningful to patients and caregivers.^22^

Investigators have proposed weight gain during treatment as a surrogate marker of longer survival in patients with NSCLC.^23^ The major determinants of weight loss versus weight gain may be tumour status and treatment‐related side effects, and weight changes are likely to affect a patient's quality of life.^24^ In the current analyses, a range of baseline characteristics, including disease stage, ECOG performance status, BMI, age, sex, and race, were associated with weight gain and survival, generally consistent with previous reports in patients with NSCLC.^17^, ^18^ Separate interaction analyses demonstrated that neither age nor tumour response had a significant impact on survival independent of weight gain. Changes in weight during treatment are captured as part of routine clinic monitoring and are potentially important for clinical study design. Further research is warranted to determine if weight changes correlate with molecular markers of cachexia.^24^

As a retrospective, post hoc analysis of pooled clinical trial data, this study has inherent design and statistical limitations that impact interpretation. The current analyses evaluated potential associations between weight gain and survival but did not assess causality. The possibility that effective cancer treatment might reverse cachexia has implications in cachexia research. Results from a small study showed that some patients with advanced NSCLC experienced either an increase or stabilisation of muscle mass during treatment with chemotherapy, and the investigators postulated that effective cancer treatment might inhibit mechanisms promoting cachexia.^25^ If data for pre‐treatment weight measurements had been available in the current study, comparison of the pre‐ and post‐treatment weight trajectories might have provided information relevant to their hypothesis. Moreover, this study did not include an evaluation of contemporary treatment options, such as immune checkpoint inhibitors, which may have a more favourable weight loss trajectory.

In conclusion, weight gain during treatment was observed in a subset of patients with advanced NSCLC and was associated with a reduced risk of disease progression and death, independent of tumour response per RECIST. The survival benefit was comparable for weight gain >0%, >2.5%, and >5.0%, suggesting that any weight gain may be associated with improved outcomes and may be useful in informing endpoint selection in interventional cancer cachexia studies.

## Conflict of interest statement

E. J. Roeland has served on scientific advisory boards for Napo Pharmaceuticals, Care4ward, Actimed Therapeutics, Meter Health, Alerion, and Takeda; as a consultant for Veloxis Therapeutics and BYOMass; and as a member of a data safety monitoring board for Enzychem Lifesciences. F. J. Fintelmann receives research support from Pfizer and serves as a consultant and speaker for Boston Scientific. L. Tarasenko, E. Whalen and R. Yang are full‐time employees and shareholders of Pfizer. R. A. Calle and F. Hilton were employees of Pfizer at the time of study execution and are Pfizer shareholders. P. D. Bonomi has received honoraria from Pfizer and Helsinn for participation in advisory boards.

## Supporting information


**Table S1.** Baseline Covariates Associated With Weight Change: Stepwise Logistic Regression.
**Table S2.** Baseline Covariates Associated With Overall Survival: Stepwise Cox Proportional Hazards Regression.
**Table S3.** Baseline Covariates Associated With Overall Survival by Weight Gain: Stepwise Cox Proportional Hazards Regression.
**Table S4.** Baseline Covariates Associated With Overall Survival by Weight Gain and Objective Response: Stepwise Cox Proportional Hazards Regression.
**Table S5.** Baseline Covariates Associated With Progression‐Free Survival: Stepwise Cox Proportional Hazards Regression.
**Table S6.** Baseline Covariates Associated With Progression‐Free Survival by Weight Gain: Stepwise Cox Proportional Hazards Regression.
**Table S7.** Baseline Covariates Associated With Progression‐Free Survival by Weight Gain and Objective Response: Stepwise Cox Proportional Hazards Regression, Model I (All Categorical Plus Time‐Dependent Weight Gain and Time‐Dependent Objective Response).

## Data Availability

Upon request, and subject to review, Pfizer will provide the data that support the findings of this study. Subject to certain criteria, conditions and exceptions, Pfizer may also provide access to the related individual de‐identified participant data. See https://www.pfizer.com/science/clinical‐trials/trial‐data‐and‐results for more information.
