# Supplementary material for: The relationship between weight gain during chemotherapy and outcomes in patients with advanced non‐small cell lung cancer
Source: J Cachexia Sarcopenia Muscle. 2024 Mar 11;15(3):1030–40. doi: 10.1002/jcsm.13426 (PMC11154746; doi:10.1002/jcsm.13426)
Supplement: Supplementary file 1 — Table S1. Baseline Covariates Associated With Weight Change: Stepwise Logistic Regression. Table S2. Baseline Covariates Associated With Overall Survival: Stepwise Cox Proportional Hazards Regression. Table S3. Baseline Covariates Associated With Overall Survival by Weight Gain: Stepwise Cox Proportional Hazards Regression. Table S4. Baseline Covariates Associated With Overall Survival by Weight Gain and Objective Response: Stepwise Cox Proportional Hazards Regression. Table S5. Baseline Covariates Associated With Progression‐Free Survival: Stepwise Cox Proportional Hazards Regression. Table S6. Baseline Covariates Associated With Progression‐Free Survival by Weight Gain: Stepwise Cox Proportional Hazards Regression. Table S7. Baseline Covariates Associated With Progression‐Free Survival by Weight Gain and Objective Response: Stepwise Cox Proportional Hazards Regression, Model I (All Categorical Plus Time‐Dependent Weight Gain and Time‐Dependent Objective Response). [file JCSM-15-1030-s001.docx]

**Supplemental Material**

**Supplemental Table 1.** Baseline Covariates Associated With Weight Change: Stepwise Logistic Regression

|  | **Model I^a^** | | | **Model II^b^** | | |
| --- | --- | --- | --- | --- | --- | --- |
| **Covariate** | **> 0% weight gain** | **> 2.5% weight gain** | **> 5.0% weight gain** | **> 0% weight gain** | **> 2.5% weight gain** | **> 5.0% weight gain** |
| Age (<65 years vs. ≥65 years) | OR = 1.377 (1.058, 1.792)  <65 years more likely to gain >0.0% weight | OR = 1.727 (1.260, 2.367)  <65 years more likely to gain >2.5% weight | OR = 1.632 (1.056, 2.520)  <65 years more likely to gain >5% weight | Age has a non-linear relationship with >0% weight gain (ie, as age increases up to about 65 years, weight increases, but after 65 years, weight decreases) | OR = 0.972 (0.957, 0.987)  Younger more likely to gain >2.5% weight | OR = 0.970 (0.950, 0.991)  Younger more likely to gain >5% weight |
| Sex (Male vs. Female) |  |  | OR = 0.610 (0.380, 0.979)  Males more likely to gain >5% weight |  |  | OR = 0.609 (0.378, 0.983)  Males more likely to gain weight |
| Baseline BMI (< 20 vs*.* ≥ 20 kg/m^2^) |  |  | OR = 2.193 (1.363, 3.528)  BMI <20 kg/m^2^ more likely to gain >5% weight |  | OR = 0.951 (0.919, 0.985)  Lower BMI more likely to gain >2.5% weight | OR = 0.903 (0.858, 0.950)  Lower BMI more likely to gain >5% weight |
| Race (Asian vs. non-Asian) | OR = 1.777 (1.268, 2.488)  Asians more likely to gain >0% weight | OR = 1.567 (1.086, 2.261)  Asians more likely to gain >2.5% weight |  | OR = 1.726 (1.228, 2.425)  Asians more likely to gain >0% weight |  |  |
| Baseline ECOG PS (0 vs. 1) | OR = 1.478 (1.135, 1.926)  ECOG 0 more likely to gain >0% weight |  |  | OR = 1.446 (1.109, 1.885)  ECOG 0 more likely to gain >0% weight |  |  |
| Baseline Stage of Disease (IIIB vs. IV) | OR = 1.829 (1.230, 2.718)  Stage IIIB more likely to gain >0% weight |  | OR = 1.846 (1.080, 3.154)  Stage IIIB more likely to gain >5% weight | OR = 1.821 (1.223, 2.712)  Stage IIIB more likely to gain >0% weight |  | OR = 1.868 (1.086, 3.216)  Stage IIIB more likely to gain >5% weight |

NOTE. Data shown as OR (95% CI).

Abbreviations: BMI, body mass index; CI, confidence interval; ECOG PS, Eastern Cooperative Oncology Group performance score; OR, odds ratio.

^a^ Model I included all covariates as categorical: age (<65 years, ≥65 years), sex (male, female), baseline BMI (<20 kg/m^2^, ≥20 kg/m^2^), race (Asian, non-Asian), smoking (never, ever), baseline ECOG PS (0, 1), adenocarcinoma (yes, no), and disease stage (IIIB, IV).

^b^ Model II included the covariates age, baseline BMI, and baseline weight as continuous variables; and sex (male, female), race (Asian, non-Asian), smoking (never, ever), baseline ECOG PS (0, 1), adenocarcinoma (yes, no), and disease stage (IIIB, IV) as categorical variables.

**Supplemental Table 2.** Baseline Covariates Associated With Overall Survival: Stepwise Cox Proportional Hazards Regression

|  | **Model I^a^** | **Model II^b^** |
| --- | --- | --- |
| **Covariate** | **Overall survival from 1^st^ day of treatment** | **Overall survival from 1^st^ day of treatment** |
| Sex (Male vs. Female) | 1.271 (1.065, 1.517)  Males have higher risk of death | 1.280 (1.073, 1.528)  Males have higher risk of death |
| Baseline BMI (< 20 vs. ≥ 20 kg/m^2^) | 1.408 (1.151, 1.723)  BMI < 20 kg/m^2^ have higher risk of death | 0.957 (0.940, 0.975)  Lower BMI increases risk of death |
| ­Race (Asian vs. Non-Asian) |  | 0.802 (0.644, 0.998)  Asians have lower risk of death |
| Smoking (Never vs. Ever) | 0.676 (0.528, 0.865)  Non-smokers have lower risk of death | 0.713 (0.553, 0.921)  Non-smokers have lower risk of death |
| Baseline ECOG PS (0 vs. 1) | 0.831 (0.712, 0.970)  ECOG 0 has lower risk of death | 0.831 (0.712, 0.970)  ECOG 0 has lower risk of death |
| Baseline Stage of disease (IIIB vs. IV) | 0.727 (0.569, 0.930)  Stage IIIB has lower risk of death | 0.733 (0.573, 0.937)  Stage IIIB has lower risk of death |

NOTE. Data shown as HR (95% CI).

Abbreviations: BMI, body mass index; CI, confidence interval; ECOG PS, Eastern Cooperative Oncology Group performance score; HR, hazard ratio.

^a^ Model I included all covariates as categorical: age (<65 years, ≥65 years), sex (male, female), baseline BMI (<20 kg/m^2^, ≥20 kg/m^2^), race (Asian, non-Asian), smoking (never, ever), baseline ECOG PS (0, 1), adenocarcinoma (yes, no), and disease stage (IIIB, IV).

^b^ Model II included the covariates age, baseline BMI, and baseline weight as continuous variables; and sex (male, female), race (Asian, non-Asian), smoking (never, ever), baseline ECOG PS (0, 1), adenocarcinoma (yes, no), and disease stage (IIIB, IV) as categorical variables.

**Supplemental Table 3.** Baseline Covariates Associated With Overall Survival by Weight Gain: Stepwise Cox Proportional Hazards Regression

|  | **Model I^a^** | | | | | **Model II^b^** | | | | |
| --- | --- | --- | --- | --- | --- | --- | --- | --- | --- | --- |
| **Covariate** | **> 0% weight gain** | **> 2.5% weight gain** | **> 5.0% weight gain** | **Percentage weight gain at each time** |  | **> 0% weight gain** | **> 2.5% weight gain** | **> 5.0% weight gain** | **Percentage weight gain at each time** |  |
| Weight Gain | 0.660 (0.567, 0.767)  >0% weight gain reduces risk of death | 0.683 (0.570, 0.818)  >2.5% weight gain reduces risk of death | 0.703 (0.548, 0.901)  >5% weight gain reduces risk of death | 0.924 (0.909. 0.938) Percentage gain in weight reduces risk of death |  | 0.638 (0.549, 0.742)  >0% weight gain reduces risk of death | 0.648 (0.541, 0.778)  >2.5% weight gain reduces risk of death | 0.677 (0.528, 0.868)  >5% weight gain reduces risk of death | 0.922 (0.907, 0.936)  Percentage gain in weight reduces risk of death |  |
| Age (<65 years vs. ≥65 years) |  |  |  | 1.192 (1.023, 1.388)  Age <65 years increases risk of death |  |  |  |  |  |  |
| Sex (Male vs. Female) | 1.272 (1.065, 1.519)  Males have increased risk of death | 1.281 (1.073, 1.530)  Males have increased risk of death | 1.292 (1.082, 1.543)  Males have increased risk of death | 1.342 (1.124, 1.602)  Males have increased risk of death |  | 1.246 (1.044, 1.486)  Males have increased risk of death | 1.265 (1.061, 1.509)  Males have increased risk of death | 1.300 (1.089, 1.552)  Males have increased risk of death | 1.339 (1.123, 1.598)  Males have increased risk of death |  |
| Baseline BMI (< 20 vs. ≥ 20 kg/m^2^) | 1.475 (1.206, 1.804)  BMI <20 kg/m^2^ increases risk of death | 1.443 (1.179, 1.766)  BMI <20 kg/m^2^ increases risk of death | 1.467 (1.196, 1.798)  BMI <20 kg/m^2^ increases risk of death | 1.431 (1.169, 1.751)  BMI <20 kg/m^2^ increases risk of death |  | 0.955 (0.938, 0.972)  Lower BMI increases risk of death | 0.955 (0.938, 0.973)  Lower BMI increases risk of death | 0.953 (0.935, 0.971)  Lower BMI increases risk of death | 0.947 (0.930, 0.965)  Lower BMI increases risk of death |  |
| Race (Asian vs. Non-Asian) |  |  |  |  |  |  |  | 0.799 (0.642, 0.995)  Asians have lower risk of death | 0.758 (0.609, 0.945)  Asians have lower risk of death |  |
| Smoking (Never vs. Ever) | 0.680 (0.530, 0.871)  Never smoking reduces risk of death | 0.668 (0.521, 0.855)  Never smoking reduces risk of death | 0.666 (0.520, 0.853)  Never smoking reduces risk of death | 0.648 (0.506, 0.830)  Never smoking reduces risk of death |  | 0.658 (0.514, 0.842)  Never smoking reduces risk of death | 0.654 (0.511, 0.837)  Never smoking reduces risk of death | 0.701 (0.543, 0.905)  Never smoking reduces risk of death | 0.686 (0.532. 0.884)  Never smoking reduces the risk of death |  |
| Baseline ECOG PS (0 vs. 1) |  | 0.846 (0.724, 0.988)  ECOG 0 reduces risk of death | 0.830 (0.711, 0.969)  ECOG 0 reduces risk of death |  |  |  | 0.849 (0.727, 0.991)  ECOG 0 reduces risk of death | 0.829 (0.710, 0.968)  ECOG 0 reduces risk of death |  |  |
| Baseline Stage of Disease (IIIB vs. IV) | 0.770 (0.602, 0.985)  Stage IIIB reduces risk of death | 0.742 (0.580, 0.949)  Stage IIIB reduces risk of death | 0.748 (0.584, 0.957)  Stage IIIB reduces risk of death | 0.775 (0.606, 0.991)  Stage IIIB reduces risk of death |  |  | 0.754 (0.589, 0.964)  Stage IIIB reduces risk of death | 0.756 (0.590, 0.967)  Stage IIIB reduces risk of death |  |  |

NOTE. Data shown as HR (95% CI).

Abbreviations: BMI, body mass index; CI, confidence interval; ECOG PS, Eastern Cooperative Oncology Group performance score; HR, hazard ratio.

^a^ Model I included all covariates as categorical: age (<65 years, ≥65 years), sex (male, female), baseline BMI (<20 kg/m^2^, ≥20 kg/m^2^), race (Asian, non-Asian), smoking (never, ever), baseline ECOG PS (0, 1), adenocarcinoma (yes, no), and disease stage (IIIB, IV).

^b^ Model II included the covariates age, baseline BMI, and baseline weight as continuous variables; and sex (male, female), race (Asian, non-Asian), smoking (never, ever), baseline ECOG PS (0, 1), adenocarcinoma (yes, no), and disease stage (IIIB, IV) as categorical variables.

**Supplemental Table 4.** Baseline Covariates Associated With Overall Survival by Weight Gain and Objective Response: Stepwise Cox Proportional Hazards Regression

|  |  | **Model I^a^** |  |  | **Model II^b^** |  |
| --- | --- | --- | --- | --- | --- | --- |
| **Covariate** | **> 0% weight gain** | **> 2.5% weight gain** | **> 5.0% weight gain** | **> 0% weight gain** | **> 2.5% weight gain** | **> 5.0% weight gain** |
| Objective Response | 0.453 (0.381, 0.539)  Responders have decreased risk of death | 0.455 (0.382, 0.541)  Responders have decreased risk of death | 0.447 (0.376, 0.532)  Responders have decreased risk of death | 0.456 (0.383, 0.543)  Responders have decreased risk of death | 0.463 (0.389, 0.552)  Responders have decreased risk of death | 0.453 (0.380, 0.538)  Responders have decreased risk of death |
| Weight Gain | 0.706 (0.606, 0.821)  >0% weight gain reduces risk of death | 0.762 (0.635, 0.913)  >2.5% weight gain reduces risk of death | 0.774 (0.603, 0.993)  >5% weight gain reduces risk of death | 0.684 (0.588, 0.797)  >0% weight gain reduces risk of death | 0.731 (0.608, 0.878)  >2.5% weight gain reduces risk of death | 0.751 (0.585, 0.964)  >5% weight gain reduces risk of death |
| Sex (Male vs. Female) | 1.213 (1.016, 1.449)  Males have increased risk of death | 1.231 (1.031, 1.470)  Males have increased risk of death | 1.223 (1.024, 1.460)  Males have increased risk of death |  | 1.221 (1.024, 1.456)  Males have increased risk of death | 1.209 (1.014, 1.442)  Males have increased risk of death |
| Baseline BMI (< 20 vs. ≥ 20 kg/m^2^) | 1.391 (1.137, 1.701)  BMI <20 kg/m^2^ increases risk of death | 1.372 (1.122, 1.679)  BMI <20 kg/m^2^ increases risk of death | 1.369 (1.117, 1.678)  BMI <20 kg/m^2^ increases risk of death | 0.963 (0.946, 0.980)  Lower BMI increases risk of death | 0.962 (0.945, 0.980)  Lower BMI increases risk of death | 0.964 (0.947, 0.982)  Lower BMI increases risk of death |
| Smoking (Never vs. Ever) | 0.682 (0.531, 0.876)  Never smoking reduces risk of death | 0.672 (0.524, 0.863)  Never smoking reduces risk of death | 0.670 (0.521, 0.860)  Never smoking reduces risk of death | 0.623 (0.490, 0.793)  Never smoking reduces risk of death | 0.664 (0.518, 0.853)  Never smoking reduces risk of death | 0.660 (0.514, 0.847)  Never smoking reduces risk of death |
| Baseline ECOG PS (0 vs. 1) |  |  | 0.852 (0.730, 0.995)  ECOG 0 reduces risk of death |  |  | 0.851 (0.729, 0.994)  ECOG 0 reduces risk of death |
| Adenocarcinoma (Yes vs. No) | 0.806 (0.671, 0.969)  Having adenocarcinoma reduces risk of death | 0.796 (0.663, 0.957)  Having adenocarcinoma reduces risk of death | 0.788 (0.656, 0.947)  Having adenocarcinoma reduces risk of death | 0.792 (0.660, 0.951)  Having adenocarcinoma reduces risk of death | 0.805 (0.669, 0.967)  Having adenocarcinoma reduces risk of death | 0.795 (0.662, 0.956)  Having adenocarcinoma reduces risk of death |
| Baseline Stage of Disease (IIIB vs. IV) | 0.767 (0.599, 0.982)  Stage IIIB reduces risk of death | 0.743 (0.580, 0.951)  Stage IIIB reduces risk of death | 0.742 (0.580, 0.950)  Stage IIIB reduces risk of death |  | 0.754 (0.589, 0.965)  Stage IIIB reduces risk of death | 0.753 (0.588, 0.964)  Stage IIIB reduces risk of death |

NOTE. Data shown as HR (95% CI).

Abbreviations: BMI, body mass index; CI, confidence interval; ECOG PS, Eastern Cooperative Oncology Group performance score; HR, hazard ratio.

^a^ Model I included all covariates as categorical: age (<65 years, ≥65 years), sex (male, female), baseline BMI (<20 kg/m^2^, ≥20 kg/m^2^), race (Asian, non-Asian), smoking (never, ever), baseline ECOG PS (0, 1), adenocarcinoma (yes, no) and disease stage (IIIB, IV).

^b^ Model II included the covariates age, baseline BMI, and baseline weight as continuous variables; and sex (male, female), race (Asian, non-Asian), smoking (never, ever), baseline ECOG PS (0, 1), adenocarcinoma (yes, no) and disease stage (IIIB, IV) as categorical variables.

**Supplemental Table 5.** Baseline Covariates Associated With Progression-Free Survival: Stepwise Cox Proportional Hazards Regression

|  | **Model I^a^** | **Model II^b^** |
| --- | --- | --- |
| **Covariate** | **Progression-free survival from 1^st^ day of treatment** | **Progression-free survival from 1^st^ day of treatment** |
| Baseline BMI (< 20 vs. ≥ 20 kg/m^2^) |  | 0.974 (0.958, 0.990)  Lower BMI increases risk of progression |
| Race (Asian vs. Non-Asian) | 1.340 (1.111, 1.615)  Asians have higher risk of progression | 1.245 (1.028, 1.508)  Asians have higher risk of progression |
| Smoking (Never vs. Ever) | 0.739 (0.598, 0.912)  Non-smokers have lower risk of progression | 0.758 (0.614, 0.936)  Non-smokers have lower risk of progression |
| Baseline ECOG PS (0 vs. 1) | 0.792 (0.683, 0.918)  ECOG 0 has lower risk of progression | 0.808 (0.697, 0.936)  ECOG 0 has lower risk of progression |
| Baseline Stage of disease (IIIB vs. IV) | 0.745 (0.594, 0.934)  Stage IIIB has lower risk of progression | 0.751 (0.599, 0.941)  Stage IIIB has lower risk of progression |

NOTE. Data shown as HR (95% CI).

Abbreviations: CI, confidence interval; ECOG PS, Eastern Cooperative Oncology Group performance score; HR, hazard ratio.

^a^ Model I included all covariates as categorical: age (<65 years, ≥65 years), sex (male, female), baseline BMI (<20 kg/m^2^, ≥20 kg/m^2^), race (Asian, non-Asian), smoking (never, ever), baseline ECOG PS (0, 1), adenocarcinoma (yes, no) and disease stage (IIIB, IV).

^b^ Model II included the covariates age, baseline BMI, and baseline weight as continuous variables; and sex (male, female), race (Asian, non-Asian), smoking (never, ever), baseline ECOG PS (0, 1), adenocarcinoma (yes, no) and disease stage (IIIB, IV) as categorical variables.

**Supplemental Table 6.** Baseline Covariates Associated With Progression-Free Survival by Weight Gain: Stepwise Cox Proportional Hazards Regression

|  | **Model I^a^** | | | | **Model II^b^** | | | | |
| --- | --- | --- | --- | --- | --- | --- | --- | --- | --- |
| **Covariate** | **> 0% weight gain** | **> 2.5% weight gain** | **> 5.0% weight gain** | **Percentage weight gain at each time** | | **> 0% weight gain** | **> 2.5% weight gain** | **> 5.0% weight gain** | **Percentage weight gain at each time** |
| Weight Gain | 0.732 (0.633, 0.846)  >0% weight gain reduces risk of progression | 0.751 (0.634, 0.889)  >2.5% weight gain reduces risk of progression |  | 0.950 (0.936, 0.964)  Percentage gain in weight reduces risk of death | | 0.718 (0.621, 0.830)  >0% weight gain reduces risk of progression | 0.721 (0.608, 0.856)  >2.5% weight gain reduces risk of progression |  | 0.946 (0.932, 0.960)  Percentage gain in weight reduces risk of death |
| Age (< 65 years vs. ≥ 65 years) | 1.163 (1.007, 1.343)  Age <65 have increased risk of progression | 1.160 (1.004, 1.340)  Age <65 have increased risk of progression |  | 1.171 (1.014, 1.351)  Age <65 have increased risk of progression | | 0.992 (0.984, 0.999)  Younger age increases risk of progression | 0.992 (0.984, 1.000)  Younger age increases risk of progression |  | 0.992 (0.984, 0.999)  Younger age increases risk of progression |
| Baseline BMI (< 20 vs. ≥ 20 kg/m^2^) |  |  |  |  | | 0.973 (0.957, 0.990)  Lower BMI increases risk of progression | 0.972 (0.955, 0.988)  Lower BMI increases risk of progression | 0.972 (0.955, 0.988)  Lower BMI increases risk of progression | 0.966 (0.950, 0.982)  Lower BMI increases risk of progression |
| Smoking (Never vs. Ever) | 0.729 (0.591, 0.900)  Never smoking reduces risk of progression | 0.717 (0.581, 0.887)  Never smoking reduces risk of progression | 0.733 (0.594, 0.906)  Never smoking reduces risk of progression | 0.717 (0.581, 0.885)  Never smoking reduces risk of progression | | 0.746 (0.604, 0.921)  Never smoking reduces risk of progression | 0.734 (0.594, 0.907)  Never smoking reduces risk of progression | 0.753 (0.609, 0.930)  Never smoking reduces risk of progression | 0.762 (0.622, 0.932)  Never smoking reduces risk of progression |
| Race (Asian vs. non-Asian) | 1.407 (1.166, 1.698)  Asians have increased risk of progression | 1.384 (1.147, 1.669)  Asians have increased risk of progression | 1.343 (1.114, 1.620)  Asians have increased risk of progression | 1.333 (1.106, 1.607)  Asians have increased risk of progression | | 1.298 (1.070, 1.573)  Asians have increased risk of progression | 1.272 (1.050, 1.542)  Asians have increased risk of progression | 1.241 (1.024, 1.503)  Asians have increased risk of progression |  |
| Baseline ECOG PS (0 vs. 1) | 0.816 (0.703, 0.946)  ECOG 0 reduces risk of progression | 0.799 (0.689, 0.927)  ECOG 0 reduces risk of progression | 0.794 (0.685, 0.920)  ECOG 0 reduces risk of progression | 0.811 (0.699, 0.940)  ECOG 0 reduces risk of progression | | 0.831 (0.716, 0.965)  ECOG 0 reduces risk of progression | 0.817 (0.704, 0.948)  ECOG 0 reduces risk of progression | 0.812 (0.700, 0.941)  ECOG 0 reduces risk of progression | 0.841 (0.725, 0.975)  ECOG 0 reduces risk of progression |
| Baseline Stage of Disease (IIIB vs. IV) | 0.764 (0.610, 0.959)  Stage IIIB reduces risk of progression | 0.760 (0.606, 0.953)  Stage IIIB reduces risk of progression | 0.753 (0.600, 0.945)  Stage IIIB reduces risk of progression | 0.778 (0.621, 0.976)  Stage IIIB reduces risk of progression | | 0.777 (0.620, 0.974)  Stage IIIB decreases risk of progression | 0.775 (0.618, 0.972)  Stage IIIB decreases risk of progression | 0.763 (0.609, 0.957)  Stage IIIB decreases risk of progression |  |

NOTE. Data shown as HR (95% CI).

Abbreviations: CI, confidence interval; ECOG PS, Eastern Cooperative Oncology Group performance score; HR, hazard ratio.

^a^ Model I included all covariates as categorical: age (<65 years, ≥65 years), sex (male, female), baseline BMI (<20 kg/m^2^, ≥20 kg/m^2^), race (Asian, non-Asian), smoking (never, ever), baseline ECOG PS (0, 1), adenocarcinoma (yes, no) and disease stage (IIIB, IV).

^b^ Model II included the covariates age, baseline BMI, and baseline weight as continuous variables; and sex (male, female), race (Asian, non-Asian), smoking (never, ever), baseline ECOG PS (0, 1), adenocarcinoma (yes, no) and disease stage (IIIB, IV) as categorical variables.

**Supplemental Table 7.** Baseline Covariates Associated With Progression-Free Survival by Weight Gain and Objective Response: Stepwise Cox Proportional Hazards Regression, Model I (All Categorical Plus Time-Dependent Weight Gain and Time-Dependent Objective Response)

|  | **Model I^a^** | | | | | **Model II^b^** | | |
| --- | --- | --- | --- | --- | --- | --- | --- | --- |
| **Covariate** | | **> 0% weight gain** | **> 2.5% weight gain** | **> 5.0% weight gain** | **> 0% weight gain** | | **> 2.5% weight gain** | **> 5.0% weight gain** |
| Objective Response | | 0.495 (0.423, 0.580)  Responders have decreased risk of progression | 0.498 (0.425, 0.584)  Responders have decreased risk of progression | 0.489 (0.417, 0.573)  Responders have decreased risk of progression | 0.502 (0.428, 0.588)  Responders have decreased risk of progression | | 0.505 (0.430, 0.592)  Responders have decreased risk of progression | 0.495 (0.423, 0.581)  Responders have decreased risk of progression |
| Weight Gain | | 0.774 (0.671, 0.894)  >0% weight gain reduces risk of progression | 0.848 (0.716, 1.003)  >2.5% weight gain reduces risk of progression |  | 0.748 (0.647, 0.866)  >0% weight gain reduces risk of progression | | 0.826 (0.697, 0.978)  >2.5% weight gain reduces risk of progression |  |
| Age (< 65 years vs. ≥ 65 years) | |  |  |  | 0.992 (0.985, 1.000)  Younger age increases risk of progression | |  |  |
| Baseline BMI (< 20 vs. ≥ 20 kg/m^2^) | |  |  |  | 0.977 (0.961, 0.994)  Lower BMI increases risk of progression | | 0.975 (0.959, 0.992)  Lower BMI increases risk of progression | 0.976 (0.960, 0.993)  Lower BMI increases risk of progression |
| Smoking (Never vs. Ever) | | 0.700 (0.567, 0.863)  Never smoking reduces risk of progression | 0.690 (0.559, 0.852)  Never smoking reduces risk of progression | 0.695 (0.563, 0.858)  Never smoking reduces risk of progression | 0.709 (0.574, 0.876)  Never smoking reduces risk of progression | | 0.708 (0.573, 0.874)  Never smoking reduces risk of progression | 0.713 (0.577, 0.880)  Never smoking reduces risk of progression |
| Race (Asian vs. non-Asian) | | 1.416 (1.174, 1.709)  Asians have increased risk of progression | 1.382 (1.146, 1.667)  Asians have increased risk of progression | 1.363 (1.130, 1.643)  Asians have increased risk of progression | 1.324 (1.091, 1.607)  Asians have increased risk of progression | | 1.285 (1.059, 1.558)  Asians have increased risk of progression | 1.269 (1.047, 1.539)  Asians have increased risk of progression |
| Baseline ECOG PS (0 vs. 1) | | 0.821 (0.708, 0.952)  ECOG 0 reduces risk of progression | 0.806 (0.695, 0.934)  ECOG 0 reduces risk of progression | 0.797 (0.687, 0.923)  ECOG 0 reduces risk of progression | 0.828 (0.713, 0.961)  ECOG 0 reduces risk of progression | | 0.823 (0.710, 0.955)  ECOG 0 reduces risk of progression | 0.812 (0.700, 0.941)  ECOG 0 reduces risk of progression |
| Baseline Stage of Disease (IIIB vs. IV) | | 0.763 (0.609, 0.957)  Stage IIIB reduces risk of progression | 0.754 (0.601, 0.945)  Stage IIIB reduces risk of progression | 0.743 (0.593, 0.933)  Stage IIIB reduces risk of progression | 0.769 (0.613, 0.964)  Stage IIIB decreases risk of progression | | 0.761 (0.606, 0.954)  Stage IIIB decreases risk of progression | 0.750 (0.598, 0.940)  Stage IIIB decreases risk of progression |

NOTE. Data shown as HR (95% CI).

Abbreviations: CI, confidence interval; ECOG PS, Eastern Cooperative Oncology Group performance score; HR, hazard ratio.

^a^ Model I included all covariates as categorical: age (<65 years, ≥65 years), sex (male, female), baseline BMI (<20 kg/m^2^, ≥20 kg/m^2^), race (Asian, non-Asian), smoking (never, ever), baseline ECOG (0, 1), adenocarcinoma (yes, no) and disease stage (IIIB, IV).

^b^ Model II included the covariates age, baseline BMI, and baseline weight as continuous variables; and sex (male, female), race (Asian, other races non-Asian), smoking (never, ever), baseline ECOG (0, 1), adenocarcinoma (yes, no) and disease stage (IIIB, IV) as categorical variables.
